# Plasticity and regeneration of gonads in the annelid *Pristina leidyi*

**DOI:** 10.1186/s13227-016-0059-1

**Published:** 2016-10-04

**Authors:** B. Duygu Özpolat, Emily S. Sloane, Eduardo E. Zattara, Alexandra E. Bely

**Affiliations:** 1 Department of Biology, University of Maryland, College Park, MD 20742 USA; 2 Institut Jacques Monod, Paris, France; 3 Department of Biology, Indiana University, Bloomington, IN USA

**Keywords:** *Piwi*, *Vasa*, *Nanos*, Gonad regeneration, Germline, Phenotypic plasticity, Starvation, Asexual reproduction, Parental effect, Annelida

## Abstract

**Background:**

Gonads are specialized gamete-producing structures that, despite their functional importance, are generated by diverse mechanisms across groups of animals and can be among the most plastic organs of the body. Annelids, the segmented worms, are a group in which gonads have been documented to be plastic and to be able to regenerate, but little is known about what factors influence gonad development or how these structures regenerate. In this study, we aimed to identify factors that influence the presence and size of gonads and to investigate gonad regeneration in the small asexually reproducing annelid, *Pristina leidyi*.

**Results:**

We found that gonad presence and size in asexual adult *P. leidyi* are highly variable across individuals and identified several factors that influence these structures. An extrinsic factor, food availability, and two intrinsic factors, individual age and parental age, strongly influence the presence and size of gonads in *P. leidyi*. We also found that following head amputation in this species, gonads can develop by morphallactic regeneration in previously non-gonadal segments. We also identified a sexually mature individual from our laboratory culture that demonstrates that, although our laboratory strain reproduces only asexually, it retains the potential to become fully sexual.

**Conclusions:**

Our findings demonstrate that gonads in *P. leidyi* display high phenotypic plasticity and flexibility with respect to their presence, their size, and the segments in which they can form. Considering our findings along with relevant data from other species, we find that, as a group, clitellate annelids can form gonads in at least four different contexts: post-starvation refeeding, fission, morphallactic regeneration, and epimorphic regeneration. This group is thus particularly useful for investigating the mechanisms involved in gonad formation and the evolution of post-embryonic phenotypic plasticity.

**Electronic supplementary material:**

The online version of this article (doi:10.1186/s13227-016-0059-1) contains supplementary material, which is available to authorized users.

## Background

Gonads are specialized organs that produce gametes and are thus fundamentally important structures in the reproductive biology of many metazoans. However, gonad development is highly variable across groups of animals and gonads can be among the most plastic organs of the body. Gonad development is often described as occurring during a typical, specific stage of the life cycle, such as during embryogenesis, larval or juvenile development, or adult sexual maturation (depending on the species) [1, 2]. It is at this stage that gonads are first established in the life cycle of the animal. This initial formation of gonads is often considered the “normal” mode of gonad formation for a species. However, gonad presence, size, and shape can be highly plastic even after this initial gonad formation phase, and this plasticity can be manifested at several points in the life cycle of an animal [3–5].

Plasticity in gonad development is manifested in a variety of ways in animals. In many animal groups, gonads can regress, regrow, change in structure, form in asexually produced individuals, and even regenerate. In groups such as insects, nematodes, ribbon worms, and mice, gonads have been shown to atrophy during periods of food limitation or starvation, and to regrow when food intake increases again [6–9]. Some animals can undergo sex reversal, involving a change in gonad structure from one gonad type (e.g., testis) to another type (e.g., ovary), a phenomenon that occurs in a number of vertebrates (e.g., fish, reptiles) and invertebrates (e.g., insects, mollusks) [10]. In groups such as colonial sea squirts, cnidarians, and sponges, gonads can form in individuals that have formed by asexual reproduction (e.g., budding) rather than by embryogenesis [3, 4, 11]. Finally, a number of animal groups have been shown to be capable of regenerating their gonads. For example, some fish and amphibians can regenerate gonadal tissue after partial or complete removal of gonads [12–14], and some flatworms and annelids can regenerate gonads after amputation of gonad-bearing body regions [15–18]. Most of the major model systems of developmental biology have none or only a few of these properties of gonad plasticity. Consequently, gonad plasticity and regeneration remain largely understudied topics, despite the wide phylogenetic distribution of such phenomena and the clear developmental importance of these processes.

Annelids, the segmented worms, are a group of animals in which several forms of gonad plasticity have been described [19–21]. With respect to the location of gametogenesis and the presence of gonads, annelids fall broadly into two groups. In non-clitellate annelids (polychaetes), which represent the bulk of annelid diversity, gametogenesis typically occurs in most segments along the body and gonads are either not present at all or are relatively simple in form, with only a thin peritoneum encasing the germ cells [19, 22]. In species without gonads at all, gamete-forming germ cells are simply distributed along the body, often in association with parapodia (lateral appendages), and without a peritoneum encasing the germ cells. In clitellate annelids, a large annelid subclade that includes leeches and oligochaetes, gonads are restricted to only a few anterior segments and gametogenesis occurs within gonads, which are typically pear-shaped sac-like structures attached to the posterior face of a segmental septum, near the ventral nerve cord [21]. In several annelids, food availability has been shown to influence the sexualization process and starvation has been shown to cause gonad regression [21, 23]. While some annelids are hermaphrodites (i.e., bearing both female and male gonads), some have separate sexes and may go through sex reversal [24–26]. Most annelids are strictly sexual species, but some species can alternate between sexual and asexual agametic reproductive phases, the latter involving some form of reproduction by fission. In these asexual phases, gonads are formed post-embryonically and may then also regress [21, 27, 28]. Finally, many species of both polychaete and clitellate annelids are able to regenerate gonads after amputation (reviewed in [16]). For example, the polychaete *Capitella teleta* (Capitellidae) can regenerate ovaries in regenerated posterior segments [18], and among the clitellates, *Lumbricillus lineatus* (Enchytraeidae), *Enchytraeus japonensis* (Enchytraeidae), *Criodrilus lacuum* (Criodrilidae), *Rhynchelmis* sp. (Lumbriculidae), and *Stylaria lacustris* (Naididae) have been documented to regenerate gonads following either anterior and/or posterior amputation ([17, 27, 29] and works by Janda reviewed in [21]). Annelids are therefore an excellent group in which to investigate plasticity of gonads.

*Pristina leidyi* Smith (1896) (Clitellata: Naididae) is a small, freshwater annelid that is well suited to studying gonad plasticity and regeneration. This species can regenerate well and can also reproduce asexually by fission. *Pristina leidyi* is hermaphroditic and forms gonads in two consecutive anterior segments: a pair of testes in segment 6 and a pair of ovaries in segment 7 (corresponding, respectively, to segments VII and VIII in classical oligochaete segment notation, which includes the asegmental prostomium in the segment count) [30]. Although this species can reproduce sexually, and does so at characteristic times of year in nature [21, 31], most generations in the wild are asexual. The cues for initiating sexual reproduction are not yet known in this species, and laboratory-cultured strains reproduce only asexually. Interestingly, we have previously shown that even in long-term asexual laboratory cultures, asexually reproducing individuals develop gonads [32]. Although these gonads do not develop to the large size characteristic of sexually mature individuals, their presence does indicate that sexual development is ongoing in asexual individuals. This same study also found that homologs of the germline/multipotency genes [33] *piwi*, *nanos*, and *vasa* are expressed in both testes and ovaries of asexual *P. leidyi*, as well as in regions of active tissue development (i.e., the regeneration blastema, posterior growth zone, and fission zone). Because gonads are relatively small and transparent in asexual individuals, gene expression thus provides a useful way to identify these structures, which are otherwise difficult to detect [32].

*Pristina leidyi* undergoes several types of post-embryonic development during which gonads can potentially form. This species rapidly regenerates both anterior and posterior body regions following amputation, completing regeneration in only 4–5 days [34]. Following anterior amputation, *P. leidyi* regenerates a maximum of four segments. If four or fewer anterior segments are removed, the number of segments that is regenerated is the number that was removed; in this scenario the gonadal segments (segments 6 and 7) remain unchanged. However, if more than four anterior segments are removed, only four segments are regenerated and the rest of the body segments undergo morphallaxis (tissue remodeling) to take on new segment morphologies consistent with their new axial location; in this scenario, segments that had not previously been gonadal segments must take on the identity of these segments. Whether morphallaxis can lead to the establishment of gonads in this species has not been documented. Under favorable conditions, individuals continuously add new segments from a subterminal posterior growth zone near the tip of the tail and reproduce asexually by paratomic fission [34–36]. During the fission process, a zone of cell proliferation is initiated within a mid-body segment, forming what is called a fission zone. Within this fission zone, new tissues develop that form a new tail for the anterior part of the worm and a new head for the posterior part of the worm; worms then physically detach. The fission process thus forms a pair of transiently linked individuals (zooids) and, if food levels are sufficiently high, multiple fission zones can be initiated in the same individual, resulting in a chain of more than two worms. When multiple fission zones form, each additional fission zone is initiated in progressively more anterior segments, as well as in the mid-body region of the posterior-most worm. Our recent study of gonad development in *P. leidyi* showed that gonads begin to form in developing heads prior to the physical separation of zooids during fission [32]. Although this previous work showed that *P. leidyi* individuals have gonads, nothing was previously known about the factors that influence the presence and size of gonads.

In this study, we characterized gonad plasticity in the annelid *P. leidyi* by investigating how gonad presence and size are influenced by extrinsic and intrinsic factors as well as by amputation. Specifically, we assessed how gonads are influenced by nutrient availability, individual age, and parental age, and determined whether individuals can regenerate their gonads after removal of gonad-bearing segments. We assessed gonad presence and size using morphology, histology, and the expression of three germline molecular markers, homologs of *piwi*, *nanos*, and *vasa*. We show that gonad presence and size are highly plastic even among clonal individuals of the same population and are sensitive to nutritional state, individual age, and parental age. We also found that, in this species, gonads can be reestablished by morphallactic regeneration, accompanying the process of segment respecification. Reviewing our findings in the context of other studies, we conclude that clitellate annelids can form gonads post-embryonically in at least four different contexts, indicating that this group of annelids is highly plastic in gonad development.

## Methods

### Animal material

*Pristina leidyi*, originally obtained from Carolina Biological Supply, were maintained as batch cultures in glass bowls (8 in. diameter) with artificial spring water (made with artificial sea salt, diluted to 1 % seawater), with paper towel as substrate and powdered *Spirulina platensis* as food. Each bowl was fed with ~0.01 g Spirulina approximately once per week. Worms reproduce continuously by fission under these conditions. To obtain rapidly fissioning individuals that possessed multiple fission zones, batch cultures were fed a 0.07-g pulse of Spirulina 5 days prior to collection. For certain experiments, worms were maintained in batches in smaller bowls (4 in. diameter) or individually in wells of a 24-well plate with ~1.5 ml spring water and fed as indicated for the particular experiment (see sections below).

### Whole mount in situ hybridization

*In situ* hybridization was carried out as previously described [36] with several modifications as previously specified [32]. Templates for the *PRIle*-*piwi1*, *PRIle*-*vasa*, and *PRIle*-*nanos* probes were, respectively, ~1900 bp (positions 990–2968 in GenBank KM078049), ~2250 bp (positions 229–2481 in GenBank KM078051), and ~1450 bp (GenBank GQ369728.1) [32]. Following development of in situ hybridization signal, samples were mounted in 25 % PBS/75 % glycerol and imaged using a Zeiss Axioplan2 microscope, a Zeiss AxioCam HRc camera, and AxioVision (v4.8.2) software.

### EdU cell proliferation assay

To assay cell proliferation within gonads, well-fed, actively growing worms were incubated with the thymidine analog EdU using the Click-iT EdU Alexa Fluor 555 Imaging Kit (Life Technologies). Animals were incubated in a 500 µM EdU solution for 1 h, and signal development was performed according to the manufacturer’s protocol.

### Methyl green-pyronin staining

Methyl green-pyronin (MGP) was used as a general histological stain as it improves detection of internal structures in the otherwise largely transparent body of *P. leidyi*. Whole worms were fixed in 70 % EtOH overnight at room temperature, hydrated to PBSt (PBS + 1 % Tween20), stained with MGP (Sigma, #HT70; provided as a ready-to-use solution by the manufacturer) for 1–2 h at room temperature, rinsed once in 90 % EtOH and twice in 100 % EtOH, transferred to EtOH/xylenes (1:1), and finally transferred to xylenes. Samples were mounted in Permount (Fisher) and imaged as described for in situ hybridization samples.

### Effect of starvation and refeeding on gonads (Experiment 1)

To assay the effect of starvation, worms from batch cultures (8-in.-diameter bowls) that had been fed within the last 4 days were transferred to a new bowl with spring water but no food. Samples were fixed for in situ hybridization on the day the experiment started (Day 0) and after 7, 14, 21, and 28 days of starvation.

To assay the effect of refeeding, worms from a culture that had been starved for 1 month (as described above) were transferred to a new culture bowl (4 in. diameter) and fed with Spirulina approximately once a week over 18 days (0.15–0.21 mg/week). Initially, no worms had fission zones; every day after refeeding individuals in the culture bowls were monitored for fission zone formation, and as soon as any fission zones were detected, all worms were transferred to individual wells of a 24-well culture plate. Worms were then maintained individually with 1.5 ml artificial freshwater containing food (0.15–0.21 mg/week). Any offspring (detached posterior zooid) produced by the original worms were discarded in order to observe the effect of refeeding on the original (anterior) zooids. Samples were fixed at the start of refeeding (Day 0), before any fission zone was observed (Day 3), as soon as single fission zones were observed in several individuals (Day 7), and once several individuals had formed multiple fission zones (Day 18).

Samples from the starvation and refeeding experiment were processed for in situ hybridization for *PRIle*-*piwi1*, *PRIle*-*vasa*, and *PRIle*-*nanos*, with processing and signal development performed in parallel and color development time kept the same for all samples to ensure comparability. For each time point, 5–10 individuals were assayed by in situ hybridization and a representative individual was imaged.

### Effect of individual age and parental age on gonad size (Experiment 2)

Worms with relatively dark gut pigmentation, indicative of older age, were collected from a standard batch culture and placed in individual wells of a 24-well culture plate. These worms were maintained individually, fed 0.15–0.21 mg/week Spirulina, and monitored every 2 days for fission. During an initial 2-week period, the health condition of each of these individuals was assayed for fission production. Most individuals produced 1–2 offspring (which were discarded) during this time; these worms were deemed to be in good condition and were kept, being subsequently referred to as O (=old) individuals. Some individuals produced no offspring during this period; these worms were suspected of being in poor condition and were discarded. The end of this initial 2-week period was used as the start of the age effect experiment (Experiment 2).

For this experiment, original O individuals continued to be fed and monitored every 2 days. The first offspring produced by each O individual during the experiment was transferred to its own well. These offspring, referred to as Y (=young) individuals, were fed and maintained individually just like O individuals. The subsequent offspring produced by O and Y individuals (which were produced within 4 days) were collected and fixed immediately, being referred to as O-offspring and Y-offspring. O and Y worms were maintained further for 1 month, with all future offspring being discarded. After 1 month, O and Y worms were fixed. All four groups of worms (O, Y, O-offspring, Y-offspring) were processed in parallel for in situ hybridization using *PRIle*-*piwi1* probe to visualize gonads. Gonad size was assayed in 5–12 individuals per group by measuring the combined maximum cross-sectional area of one testis and one ovary (either left or right) in lateral view using Image J [37].

### Gonad regeneration after amputation (Experiment 3)

To elicit regeneration, worms were anesthetized in 50 μM nicotine in spring water and amputated anteriorly (between segments 7 and 8) and posteriorly (between segments 18 and 19) with a scalpel. Worms without visible fission zones were used as regeneration material, because the presence of fission zones can delay or inhibit regeneration [38]. Chaetae (segmental bristles) were used as visual landmarks for determining the desired amputation location; cuts were placed between the chaetae of consecutive segments. These 11-segment pieces of the mid-body (which were devoid of the gonad-bearing segments as well as the posterior growth zone) were maintained in spring water with food (0.15–0.21 mg/week Spirulina) until fixation. Specimens were fixed at several time points after amputation and processed for *PRIle*-*piwi1* in situ hybridization.

### Statistical analysis

Analyses were performed in R statistical computing environment [39] using Wilcoxon rank-sum tests. Error bars in all graphs represent standard error.

## Results

### Gonads regress upon starvation and regrow upon refeeding

We investigated whether the gonads of *P. leidyi* are sensitive to feeding regime by starving and refeeding worms over several weeks and assessing gonad expression of three germline/multipotency genes, homologs of *piwi*, *nanos*, and *vasa* (“Methods,” Experiment 1). We also scored specimens for the presence of fission zones and expression in developing regions, namely in fission zones and the posterior growth zone. Consistent with our previous study [32], we found that all three genes were expressed in gonads and actively growing tissues but we determined that our *PRIle*-*piwi1* probe typically yielded the strongest and most robust expression in the gonads; scorings and data presented below therefore emphasize results from this gene.

**Fig. 1:**
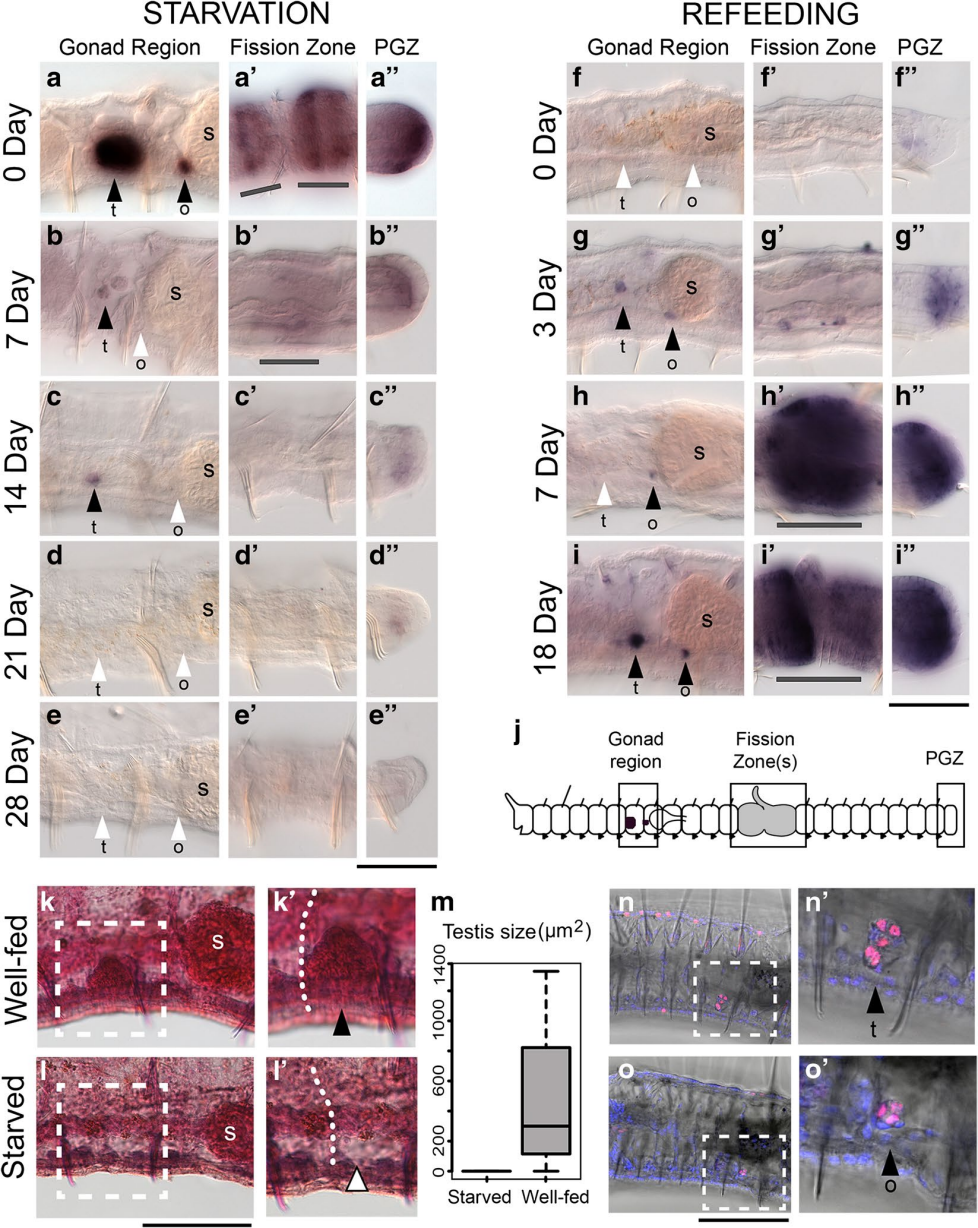
Effect of starvation and refeeding on *PRIle*-*piwi1* expression and gonad size. **a**–**i**″ *PRIle*-*piwi1* expression in the gonad region (**a**–**i**), fission zone region (**a**′–**i**′), and posterior growth zone (**a**″–**i**″) during starvation (**a**–**e**″) and refeeding (**f**–**i**″). Expression decreases and becomes undetectable in all three body regions as worms are starved over 28 days and is reestablished in all three body regions when starved worms are fed over 18 days. All images were taken at the same magnification; worms shrink when starved. (**k**–**l**′) MGP staining in the gonad region of segment 6 in well-fed and starved worms. Testes are evident in well-fed animals (*dashed box* in **k**; *black arrowhead* in **k**′) but not detectable in starved animals (*dashed box* in **l**, *white arrowhead* in **l**′). **k**′ and **l**′ are magnified views of the *boxed regions* in **k** and **l**. *Curved dotted line* in **k**′ and **l**′ indicates the position of the septum between segments 5 and 6. **m** Quantification of testis size in starved and well-fed animals. Animals starved for 1 month had no detectable gonads (*n* = 24); well-fed animals typically had gonads, but these were highly variable in size (*n* = 14). **n**–**o**′ EdU incorporation in gonads of developing head segments being formed by fission. *Magenta label* indicates proliferative (EdU-positive) cells. **n**′ and **o**′ are magnified views of the *boxed regions* in **n** and **o**. *Black arrowheads* indicate the presence of gonad expression or gonad structures; *white arrowheads* indicate the absence of such expression or structures. Fission zones are marked by *gray bars*. Anterior is *left* in this and all other figures. *t* testis, *o* ovary, *s* stomach. *Scale bars* 100 µm

We found that starvation causes a marked body-wide down-regulation of germline/multipotency gene expression and appears to cause gonad size to decrease. At the start of the experiment, well-fed worms had robust *PRIle*-*piwi1* gonad expression, displayed multiple fission zones, and had strong expression in fission zones and the posterior growth zone (Fig. 1a–a″). After just 1 week of starvation, the region of gonad expression was dramatically smaller, worms showed fewer fission zones, and expression was markedly decreased in fission zones and the posterior growth zone (Fig. 1b–b″). Expression continued to decrease through Day 14 and Day 21 of starvation (Fig. 1c–c″, d–d″) until, by Day 28, no gene expression was detected anywhere in the body (Fig. 1e–e″). Although not quantified, worms also appeared to shrink in size during the month of starvation. A similar effect of starvation on expression in the fission zone and posterior growth zone was found for *PRIle*-*nanos* and *PRIle*-*vasa* (Additional file 1: Figures S1, S2). In situ hybridization probes for other genes we have investigated also show weaker expression in less well-fed worms (B.D.O. and A.E.B., unpublished data), suggesting that this may be a general effect in *P. leidyi*, and one that warrants further study.

When worms starved for 1 month were refed, within just a few days the expression in gonads and zones of growth rapidly became detectable again, gonad size appeared to increase, and fissioning was reinitiated. By Day 3 of feeding, *PRIle*-*piwi1* was upregulated in the gonads and posterior growth zone (Fig. 1g, g″), and by Day 7, fission zones were established and expression in both fission zones and the posterior growth zone became strong (Fig. 1h′, h″). Gonad expression was restricted and variable through Day 7, with expression being limited to small clusters of cells and with most worms displaying expression in only a subset of the four gonad positions (left/right testes, left/right ovaries) (Fig. 1g, h), though a few individuals displayed expression in all four gonad positions. By Day 18 of feeding, expression in gonads was stronger and more extensive than on Day 7 and expression was consistently detected in both testes and both ovaries; expression in fission zones and posterior growth zones became stronger still at this time point as well (Fig. 1i–i″). A similar effect of refeeding on expression in the fission zone and posterior growth zone was found for *PRIle*-*nanos* and *PRIle*-*vasa* (Additional file 1: Figures S1, S2).

To determine whether cells within the gonad can undergo cell proliferation, we incubated well-fed worms with EdU. We found that in actively growing worms, cells within the gonads do proliferate. In heads developing within the fission zone, some cells in both the testes (Fig. 1n, n′) and the ovaries (Fig. 1o, o′) are positive for EdU incorporation. This indicates that at least some increase in gonad size can occur by cell proliferation within the gonad.

We confirmed that gonad size, not just gonadal gene expression, is influenced by feeding regime by using histology. Starting with a well-fed batch culture, we fixed one group of worms immediately and starved the remainder of worms for 1 month before fixation. The two groups of worms were stained with methyl green-pyronin (MGP) in parallel, and testis size (cross-sectional area of the left or right testis in lateral view) was measured (ovaries were too difficult to score due to their small size and close proximity to the dilated stomach). In well-fed individuals, testis size had a median of 299.5 µm^2^ but also showed a large range, from 0 to 1330 µm^2^ (Fig. 1m). Of these well-fed individuals, most (11/14) had testes that were histologically detectable (Fig. 1k, k′) while 3/14 had no detectable testes (we estimate we could detect testes cell clusters larger than a few cells). By contrast, none of the worms starved for 1 month (0/24) had detectable testes (Fig. 1l, l′). Thus, starvation not only causes gonad gene expression to become undetectable, but it causes the gonads themselves to disappear or shrink to a size below detectability. Overall the starvation and refeeding experiments showed that, depending on the nutritional state, gonads can regress (to the point of being histologically and molecularly undetectable) and then regrow within the same segments.

### Both individual age and parental age affect gonad size

The previous experiment indicated that food has a major effect on the presence/absence of gonads but also highlighted that worms kept under the same environmental conditions (same culture, same food availability) can have dramatically different gonad sizes (Fig. 1m). This suggests that gonad size may be influenced by one or more additional factors that are likely non-environmental. Our *P. leidyi* cultures are clonal, reproducing exclusively asexually, so genetic differences are unlikely to be one of these factors but we speculated that age could be an important factor affecting gonad size. Because of *P. leidyi*’s mode of growth and fission, any individual worm has segments of varying age. However, because most segments are formed sequentially from a posterior growth zone, the segments of an individual typically follow an anterior to posterior age gradient, with more posterior segments being younger (new head segments formed by fission are the exception, since they are younger than the segments immediately posterior to them). Therefore, when a worm fissions into two, the posterior zooid (offspring) is on average younger than the anterior zooid (parent).

**Fig. 2:**
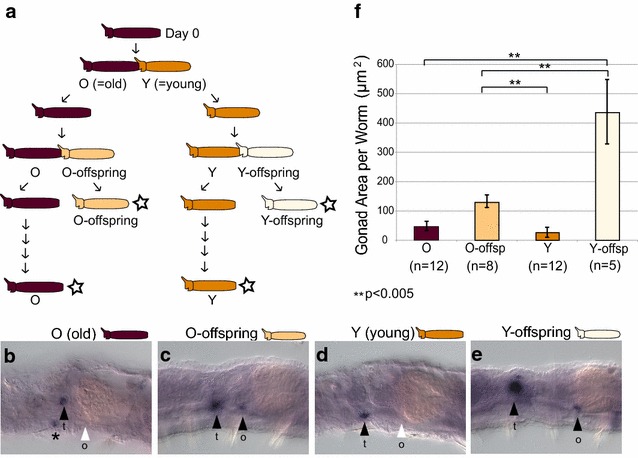
Effect of individual and parental age on gonad size. **a** Experimental setup, indicating relationship between old worms (O), young worms (Y), offspring of old worms (O-offspring), and offspring of young worms (Y-offspring). *Arrows* indicate aging and fission events. *Stars* indicate the four groups of worms that were fixed for in situ hybridization analysis. **b**–**e** Gonad region of representatives from the four groups of worms analyzed for *PRIle*-*piwi1* in situ hybridization. *Black arrowheads* indicate the presence of gonad expression; *white arrowheads* indicate the absence of gonad expression; *t* and *o* indicate testis and ovary, respectively. *Asterisk* in **b** indicates the second (right) testis that is also in focus in this image. **f** Gonad size in the four groups of worms. The oldest group (O) has significantly smaller gonads than the youngest group (Y-offspring) (*W* = 0, *p* = 0.0016), the offspring of old parents (O-offspring) have significantly smaller gonads than the offspring of young parents (Y-offspring) (*W* = 1, *p* = 0.0031), and O-offspring have significantly larger gonads than Y individuals (which are older offspring of the same parents) (*W* = 86, *p* value = 0.0028). O and Y were not significantly different from each other (*W* = 89, *p* = 0.2991). Gonad size was measured as the combined area of one testis and one ovary (either left or right) for each worm

We tested two age-related hypotheses: (1) The age of an individual worm can influence gonad size, and (2) the age of an individual’s “parent” (the anterior worm from which the individual was produced by fission) can influence gonad size. We tested the first by comparing gonad size in old individuals to that of young offspring and tested the second by comparing gonad size of offspring from old parents to that of offspring from young parents (see “Methods,” Experiment 2; Fig. 2a).

We found that both individual age and parental age had a significant effect on gonad size (Fig. 2b–f). The youngest individuals in our experiment had significantly larger gonads than the oldest individuals (Y-offspring > O individuals; *p* < 0.005; Fig. 2f), younger offspring had significantly larger gonads than older offspring of the same parents (O-offspring > Y individuals; *p* < 0.005), and offspring of younger parents had significantly larger gonads than the offspring of older parents (Y-offspring > O-offspring; *p* < 0.005). Although overall worm size was not quantified, individuals in our different categories were all roughly similar in size and gonad size did not simply scale with worm size. In fact, recent offspring (O-offspring and Y-offspring) tended to be slightly smaller than older worms (O and Y), and yet these recent offspring had the largest gonads. The magnitude of gonad size variation was also very large; for example, average gonad size of Y-offspring was approximately ten times that of their aged parents (Y individuals). Although O and Y individuals (the two oldest sets of individuals in our experiment) differed in age, gonad size was not significantly different between them. Although we do not have an age estimate for O individuals (though we know they are considerably older than 1 month), we know that Y individuals were 1 month old at the time of collection. The lack of difference in gonad size between O and Y individuals therefore indicates that the dramatic age-related changes in gonad size we detected in this experiment occurred over less than 1 month. We conclude that gonad size is greatest in young individuals (newly detached posterior fission products) and, especially, in young individuals from young parents and that gonads then shrink dramatically during the first month after detachment.

### *Pristina leidyi* can establish gonads in previously non-gonadal segments by morphallactic regeneration

**Fig. 3:**
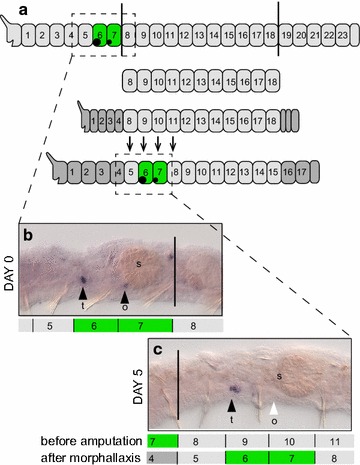
Gonad regeneration by morphallaxis. **a** Summary of amputation scheme and regeneration results for gonad regeneration. Worms were double-amputated (*black vertical lines*), removing the posterior end and 7 anterior segments. Anterior segments removed included the gonadal segments, segments 6 and 7. Worms regenerated only four head segments anteriorly by epimorphosis but gonads regenerated by morphallaxis in the segments that were previously segments 9 and 10, now segments 6 and 7. *Dark gray* regenerated segments; *Green* gonadal segments. Note the posterior shift in segment identity (numbers) after amputation. **b** Unamputated control worm with gonads revealed by *PRIle*-*piwi1* expression. **c** Double-amputated worm at 5 dpa, with *PRIle*-*piwi1* expression in gonads (testes) of the segment that was formerly segment 9 (a non-gonadal segment) but is now positioned as segment 6. *t* testis, *o* ovary, *s* stomach

The starvation-refeeding experiment showed that gonads can regress and then regrow in the same segments that previously contained gonads. To follow-up on these findings, we next asked whether *P. leidyi* can develop gonads in segments that were previously non-gonadal segments, through a process of morphallactic regeneration. In *P. leidyi* the two gonad-bearing septa (separating segments 5 and 6 and separating segments 6 and 7) can be formed during fission but not during epimorphic (blastema-based) regeneration. This is because in *P. leidyi,* although 6 segments form anteriorly by fission, a maximum of only 4 segments can be formed anteriorly by regeneration [34]. If more than 4 segments are removed, only 4 segments are regenerated (by epimorphosis) and the remaining segments undergo morphallaxis (tissue remodeling) to reestablish the appropriate segmental identities and morphologies along the body (see diagram of Fig. 3a).

Given that gonad-bearing septa do not form anew during regeneration in *P. leidyi*, we investigated whether gonads can still be reestablished in individuals following removal of gonad-bearing segments. For this experiment we chose to perform double amputations, cutting worms between segments 7 and 8 (removing both gonad-bearing segments; Fig. 3b) and again between segments 18 and 19 (removing the posterior segments and posterior growth zone), retaining an 11-segment-long piece of the mid-body (see “Methods”, Experiment 3; Fig. 3a). This generated a body fragment that was devoid of both body regions (head and tail) that exhibit high germline/multipotency gene expression [32] and therefore might be likely potential sources of new germline.

We found that by 5 days post-amputation (dpa), gonadal expression of *PRIle*-*piwi1* was established in segments that were previously non-gonadal (Fig. 3b, c). Specifically, by 5 dpa, four segments had regenerated anteriorly and segments that were originally segments 9 and 10, now positioned as segments 6 and 7, developed gonad expression. At 5 dpa, most worms had *PRIle*-*piwi1* expression in testes (segment 6). A few animals were further maintained and fed until 18 dpa; testes were consistently detected in these animals, and ovaries were detected in a few specimens as well. Specimens processed at an intermediate regeneration time point (Day 3) suggest gonad expression arises de novo; we found no evidence of *PRIle*-*piwi1* positive cells in or near the segments transforming into gonadal segments at this earlier time point. Our results demonstrate that, in *P. leidyi*, gonads can regenerate by morphallaxis of previously non-gonadal segments and suggest that establishment of testes may precede establishment of ovaries, a sequence that is common in other annelids undergoing sexualization [21].

### An asexually propagated laboratory strain of *P. leidyi* can still undergo sexualization

**Fig. 4:**
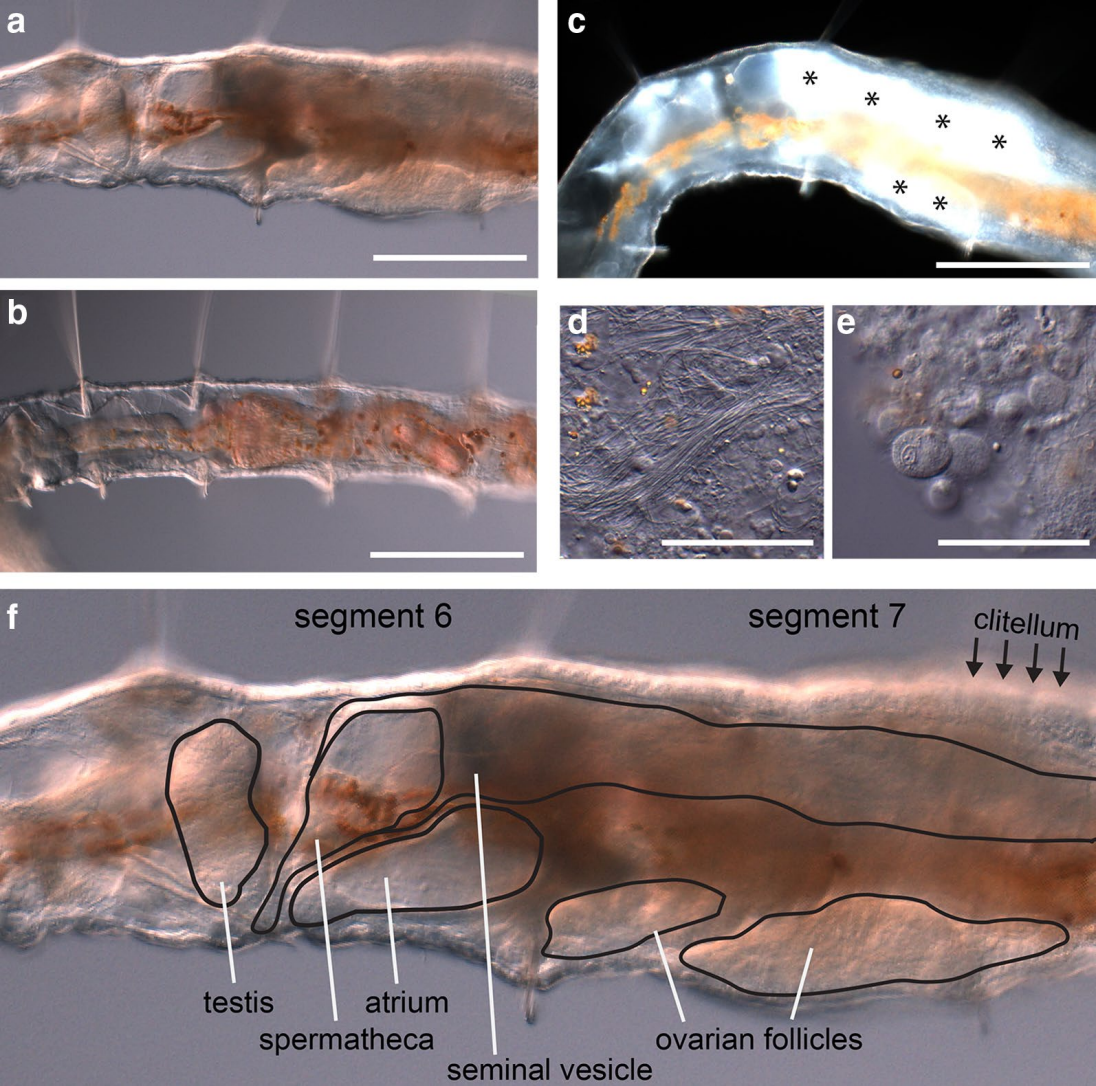
Sexually mature *P. leidyi*. **a** Sexual individual (DIC imaging). **b** Asexual individual from the same culture as **a**. **c** The same sexual individual as in **a**, imaged using darkfield, showing the gametes (appearing *white*, indicated by *asterisks*) within the body. **d**–**e** Sperm and oocytes, respectively, dissected from the worm. **f** Same image as **a**, but with sexual structures outlined and labeled. The marked structures were identified based on the anatomy of sexual naidids and other clitellates as described in [21, 65]. *Scale bars* 200 µm (**a**–**c**); 50 µm (**d**, **e**)

In natural populations of naidid species capable of fission, fission tends to be the primary mode of reproduction but sexually mature individuals can be found in the wild [21, 30, 31], typically at brief but characteristic times of year [40]. Because conditions that promote sexual maturation are unknown for *P. leidyi*, we are not able to induce sexuality and obtain fully mature individuals. However, we recently found a sexually mature individual in our laboratory cultures (Fig. 4). This individual is a rare specimen, being the only one noted in our cultures in more than a decade of culturing. However, it is significant in that it demonstrates the potential of this asexually maintained laboratory strain to become sexualized. This individual produced recognizable gametes and sexual anatomy: It made sperm and oocytes (Fig. 4c–e), formed sexual apparatus such as the spermatheca, atrium, and seminal and ovarian vesicles (Fig. 4a, f), and had a clitellum, the collar of thickened epithelium around the sexual segments (Fig. 4a, f). This singular yet unambiguous sexual individual demonstrates that this laboratory strain retains the ability to become fully sexually mature.

## Discussion

Building on a recent study showing that gonad structures form in asexually reproducing individuals of the annelid *Pristina leidyi* [32], in this study we aimed to characterize gonad plasticity and to investigate gonad regeneration in this species. We identified both extrinsic and intrinsic factors that influence the presence and size of gonad structures and found that gonads in *P. leidyi* can develop by morphallactic regeneration in previously non-gonadal segments. Furthermore, although gonad size remains relatively small in all asexual individuals, we identified a sexually mature individual from our laboratory cultures indicating that this laboratory strain of *P. leidyi* retains the potential to become fully sexual. Together, our findings demonstrate that gonads in *P. leidyi* are highly plastic with respect to their presence, their size, and their location. We find that, as a group, clitellate annelids can form gonads in at least four different contexts, making this group a particularly useful one for investigating the developmental, physiological, and molecular mechanisms involved in gonad formation.

### Nutritional status, individual age, and parental age all strongly influence gonad size in asexual *P. leidyi*

Although cultures of *P. leidyi* reproduce clonally, and thus little if any genetic variation is expected among individuals, we noted very high variation in the size of developing gonads among individuals in our cultures. Our experiments showed that gonad size is extremely sensitive to both extrinsic and intrinsic factors, specifically feeding level, individual age, and parental age (Figs. 1, 2). The functional consequence of differences in gonad size is not yet known, but a possible explanation is that this variation correlates with individuals being closer to or farther from sexual maturity. Testing this idea would require knowing the factors triggering sexual maturation, such that individuals with gonads of different sizes could be assayed for how readily or quickly they become fully sexual. Although such factors are not yet known, precluding testing this idea at this time, the identification of a sexually mature individual from our cultures indicates that sexual maturity can indeed be reached in this species even after extended periods of exclusively asexual reproduction (Fig. 4).

Starvation-induced gonad regression is known to occur in many animal groups, including several annelids [6, 9, 21, 41, 42]. When starved, *P. leidyi* individuals stop fissioning and their gonads shrink dramatically, becoming undetectable (by either in situ hybridization or histological staining) by 3–4 weeks without food. We estimate that if gonads are present at all, they must be composed of only a few cells at most and either do not express the *piwi* homolog we investigated, *PRIle*-*piwi1*, or do so at extremely low levels, below detectability by in situ hybridization. However, when starved worms are refed, they redevelop detectable gonads with strong *PRIle*-*piwi1* expression within just a few days. Cells within the gonads of well-fed worms are positive for EdU labeling, indicating that gonad growth occurs, at least in part, by cell proliferation within the gonad. In refed worms, *PRIle*-*piwi1* expression in the gonad area appears to arise de novo, since starved worms do not express detectable levels of *PRIle*-*piwi1* anywhere in the body. What the cellular source is of the reestablished gonads remains an important question to address. It is possible that a few gonad cells or stem cells persist at the gonad site and regrow the gonads when feeding is restored, or that all gonad cells disappear under starvation but, when feeding is restored, source cells are induced and/or migrate from some other location to reestablish the gonad. Recent studies in *P. leidyi* have documented migration of cells using live imaging during both regeneration [43] and fission [32], and a study in another clitellate annelid has inferred migration of germline precursor cells from snapshot data during regeneration [17]; the cell migration hypothesis for gonad establishment is thus a viable one worth investigating further. Whether the source cells of reestablished gonads express *PRIle*-*piwi1* at very low levels or not at all, and whether they express *PRIle*-*piwi1* protein, should also be investigated in future studies.

Since many animals do not produce mature gonads until they reach a certain size or age, we had expected that, if age effects were present in *P. leidyi*, older worms would be the ones to have larger gonads (on the assumption that they are closer to maturation). However, we found the exact opposite: The largest gonads are found in the youngest worms (newly fissioned posterior worms) (Fig. 2). Specifically, we found that young fission products start off with large gonads and these gonads shrink dramatically over the next few weeks. Several scenarios could account for gonad shrinkage as individuals age. Shrinkage could be mediated by environmental cues. For example, the default state of young individuals (young fission products) may be to become sexually mature (and thus to have large gonads) but then, in response to cues from the environment (which in the laboratory favor only asexual reproduction), they may invest resources preferentially into other processes rather than maintaining large gonads. The cause of shrinkage could also be due to changes in the individual’s energy budget, which is known to be dynamically allocated in this species [38]. For example, young worms might start off with the greatest energy budget, having received considerable investment from their (anterior) parent, and thus having the most energy available to invest into gonads; once these animals need to sustain themselves by feeding and invest energy into fission products themselves, energy may be better allocated into these processes rather than in maintaining large gonads. Gonad shrinkage could also result from a general decrease in body function, namely senescence, and/or a depletion of germline cells. If, for example, the cells that make the gonads of a fission product must come from the (anterior) parent worm’s gonads, successive rounds of fission might eventually deplete the anterior worm’s germline cells. Testing these hypotheses and investigating the ultimate and proximate causes of gonad shrinkage (and growth) will help elucidate mechanisms of germline plasticity.

Our study also demonstrated a strong parental effect on asexually produced offspring (Fig. 2). The offspring (posterior zooids) of young parents (anterior zooids) had significantly larger gonads than the offspring of older parents. Therefore, just as is found in sexually produced offspring of many species [44–48], and also in offspring produced via parthenogenesis [49], features of asexually produced offspring can be strongly influenced by the age of the parent. The cause(s) of this parental effect could include scenarios similar to those described above for individual-level age effects. Whether the parental effect we identified in *P. leidyi* is in some way indicative of senescence of the anterior zooid remains to be determined. Evidence of senescence has been demonstrated in several asexual invertebrates (e.g., colonial tunicates, bryozoans, hydroids in [50]), including another fissioning naidid (*Paranais litoralis*) [51], so this is a viable possibility.

### Gonads can develop in a variety of post-embryonic contexts in *P. leidyi* and in annelids more broadly

**Fig. 5:**
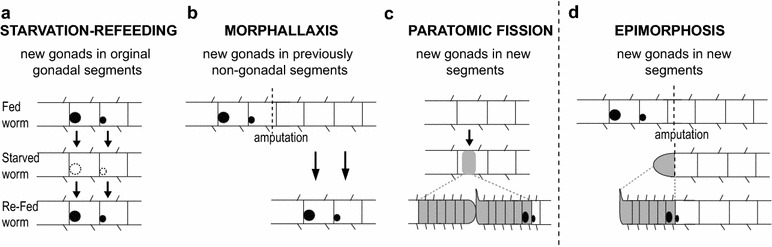
Post-embryonic contexts in which gonad development occurs in clitellate annelids. **a** In starved animals that have lost detectable gonads, gonads are reestablished upon feeding in the original gonadal segments. **b** During morphallactic regeneration, gonads develop in previously non-gonadal segments. **c** During paratomic fission, gonads arise in newly formed segments that develop within the fission zone; note that gonads (ovaries) are shown attached to the poster-most new septum (as previously suggested [32]), extending from new tissue (*gray*) into previously existing tissue. **d** During epimorphic regeneration following amputation (or fragmentation), gonads develop in newly formed segments; shown here is a scenario in which gonads form on the two posterior-most new septa made during regeneration, as seen during regeneration in a number of clitellate annelids. As in *panel* **c**, new tissue is indicated by *gray* shading

Findings from this and other studies suggest that gonads can be established in a variety of post-embryonic contexts in *P. leidyi* and in clitellate annelids more broadly (Fig. 5) [17, 27, 32, 52]. Gonads can be induced by feeding, can form by morphallaxis following amputation, can form during paratomic fission, and can develop by epimorphic regeneration. We found that the first three of these can all occur in *P. leidyi* (this study; [32]), indicating that this species has a particularly broad repertoire for establishing gonads post-embryonically; the last of these appears to not be possible in *P. leidyi* but has been described in several other annelids [17, 27, 52].

Findings from this study indicate that the presence of detectable gonads in *P. leidyi* is sensitive to feeding regime, with individuals losing detectable gonads when starved and regrowing gonads within days after refeeding (Fig. 5a). A strong effect of feeding on gonads has also been demonstrated in other annelids, such as the annelid *Enchytraeus japonensis* in which sexualization is dependent on feeding [23]. Food availability is known to influence gonad development and maintenance in a number of animals; for example, in macrostomid flatworms, gonads of starved adults shrink to below detectability [41, 42], similar to our findings in *P. leidyi*, and in insects, starved larvae form fewer ovarioles [53] and adults can resorb oocytes [9]. We therefore expect that feeding-dependent gonad formation may be widespread among annelids.

Our study also confirms that gonads can form by morphallaxis of previously non-gonadal segments following amputation in *P. leidyi* (Fig. 5b). In this species, a maximum of four segments can regenerate anteriorly; if more than four are amputated anteriorly, the normal morphology of the animal is reestablished by tissue remodeling of the remaining original segments. In this study, following the removal of seven anterior segments, we found that gonads are established in segments that were formerly non-gonadal segments (segments 9 and 10) when these segments acquire the body position typical of gonadal segments (segments 6 and 7) (Fig. 3). Gonad formation by morphallaxis has been documented in another clitellate annelid [17], but otherwise remains poorly studied. Given that morphallaxis of somatic body features is common in annelids [54], the possibility that gonad morphallaxis occurs more broadly among annelids should be further investigated.

In *P. leidyi*, the process of paratomic fission involves the novel formation of the six anterior-most segments, including the septum between segments 5 and 6 and the septum between segments 6 and 7; it is on these two newly formed septa (the two posterior-most septa formed within the new head) that *P. leidyi* forms gonad structures during fission (Fig. 5c). Thus, *P. leidyi* paratomy involves formation of gonads from newly developed tissues, similar to gonad formation during epimorphic regeneration (Fig. 5d). Paratomy in *P. leidyi* is thought to have evolved as a modification of the epimorphic regeneration process [34, 55]; thus, it is possible that the similarity between gonad formation during paratomy and that during epimorphic regeneration (present in other annelids, though not in *P. leidyi*) is due to the shared ancestry of these processes. Paratomy has evolved a number of times within annelids, and investigating gonad formation in additional species representing independent origins of paratomy will be needed to determine how widespread this mechanism of gonad development is in annelids.

Finally, in some annelids, though not *P. leidyi*, gonads form in segments that regenerate by epimorphosis (Fig. 5d). In *P. leidyi*, epimorphic regeneration of gonads does not occur because the maximum number of segments that can regenerate in this species (four) is less than the segment number in which gonads normally form (segments 6 and 7); thus, the gonadal segments are not regenerated epimorphically (but instead by morphallaxis of old segments). *P. leidyi* appears to be unusual in this regard, however. More typically, in clitellates, the gonadal segments, or at least the septa on which gonads will form, can regenerate; indeed, these are often the posterior-most structures that can be regenerated anteriorly [27, 54, 56, 57]. In this regard, an interesting case involves a fissioning species of *Enchytraeus*, in which individuals that develop from zygotes form gonads in segments 10 and 11, but in which a maximum of seven new segments can regenerate anteriorly. Individuals produced by fragmentation/regeneration have gonads shifted anteriorly with respect to embryonically derived individuals; gonads of these asexually produced individuals occur on the posterior-most regenerated septa, namely segments 7 and 8, instead of in segments 10 and 11 [17, 52]. Regenerating gonads through epimorphosis, that is, on newly developed septa, thus appears to be an ancestral mechanism of gonad regeneration among clitellates, and one that is retained in certain asexually reproducing species, though not *P. leidyi*.

Importantly, across these four post-embryonic contexts (feeding, morphallaxis, paratomy, and epimorphosis), gonads form in different types of segments and with different cell type associations (Fig. 5). When starved worms are refed, gonads form in original segments that previously possessed gonads; during morphallaxis, gonads form in original segments that were previously non-gonadal; and during both paratomic fission and epimorphic regeneration, gonads form in newly formed segments. Several studies in annelids, including a prior study in *P. leidyi*, have suggested that isolated *piwi*-positive cells may be involved in germline development in annelids [17, 18, 32, 58]. In particular, our previous work in *P. leidyi* suggested that a population of *piwi*-positive cells found along the ventral nerve cord appears to be migratory and involved in development of gonads during fission [32], and a study in the clitellate *Enchytraeus* similarly inferred that gonad formation is associated with migration of *piwi*-positive cells [17]. However, in this study, we found no evidence that gonad formation is associated with the presence of *piwi*-positive presumptive germ cells during refeeding and morphallaxis. Together, these differences across post-embryonic contexts suggest the possibility that gonads may form by different post-embryonic processes in annelids, a possibility that should be investigated in future studies.

## Conclusions

This study demonstrates that, in a clitellate annelid, gonads are highly plastic structures that can vary in size, can be present or absent, can form in different segments, are sensitive to extrinsic and intrinsic factors, and can form in multiple post-embryonic contexts. Whereas a few other anatomical structures have been shown in annelids to undergo morphallactic regeneration or to vary in size in response to extrinsic cues in adult individuals, the sexual organs appear to be particularly plastic. Thus, annelid gonads may be useful structures to focus on for investigating the developmental basis of phenotypic flexibility (reversible, within-individual variation) and phenotypic plasticity more broadly, phenomena increasingly recognized as being pervasive and important in development and evolution [59–64]. In future work, it will be important to identify the cues and developmental, molecular, and physiological mechanisms involved in gonad development and regression and to determine to what extent these are similar across different contexts. It will be of particular interest to identify the cell sources of post-embryonically formed gonads and specifically their germline source. Determining how gonad development during post-embryonic contexts compares to that during embryogenesis will also be of particular interest, as it should provide important insights into how gonad plasticity can evolve. Ultimately, comparative studies across different groups of annelids, and animals more broadly, will be needed to understand the evolutionary origins and diversification of plasticity of gonads.

## Additional file

10.1186/s13227-016-0059-1 Effect of starvation and refeeding on *PRIle*-*nanos* and *PRIle*-*vasa* expression.

## Abbreviation

dpa: days post-amputation
