# Supplementary material for: Plasticity and regeneration of gonads in the annelid Pristina leidyi
Source: EvoDevo. 2016 Oct 4;7:22. doi: 10.1186/s13227-016-0059-1 (PMC5051023; doi:10.1186/s13227-016-0059-1)

**Additional File 1**

**Figure S1 – Effect of starvation and refeeding on *PRIle-nanos* expression.** (A-J') *PRIle-nanos* expression in the fission zone region (A-J) and posterior growth zone (A'-J') during starvation (A-F') and refeeding (G-J'). Expression decreases and becomes undetectable in both body regions as worms are starved over 28 days and is re-established in both body regions when starved worms are fed over 18 days. Fission zones are marked by gray bars. Note that animals in A and J possess multiple fission zones. Scale bars: 100 µm.


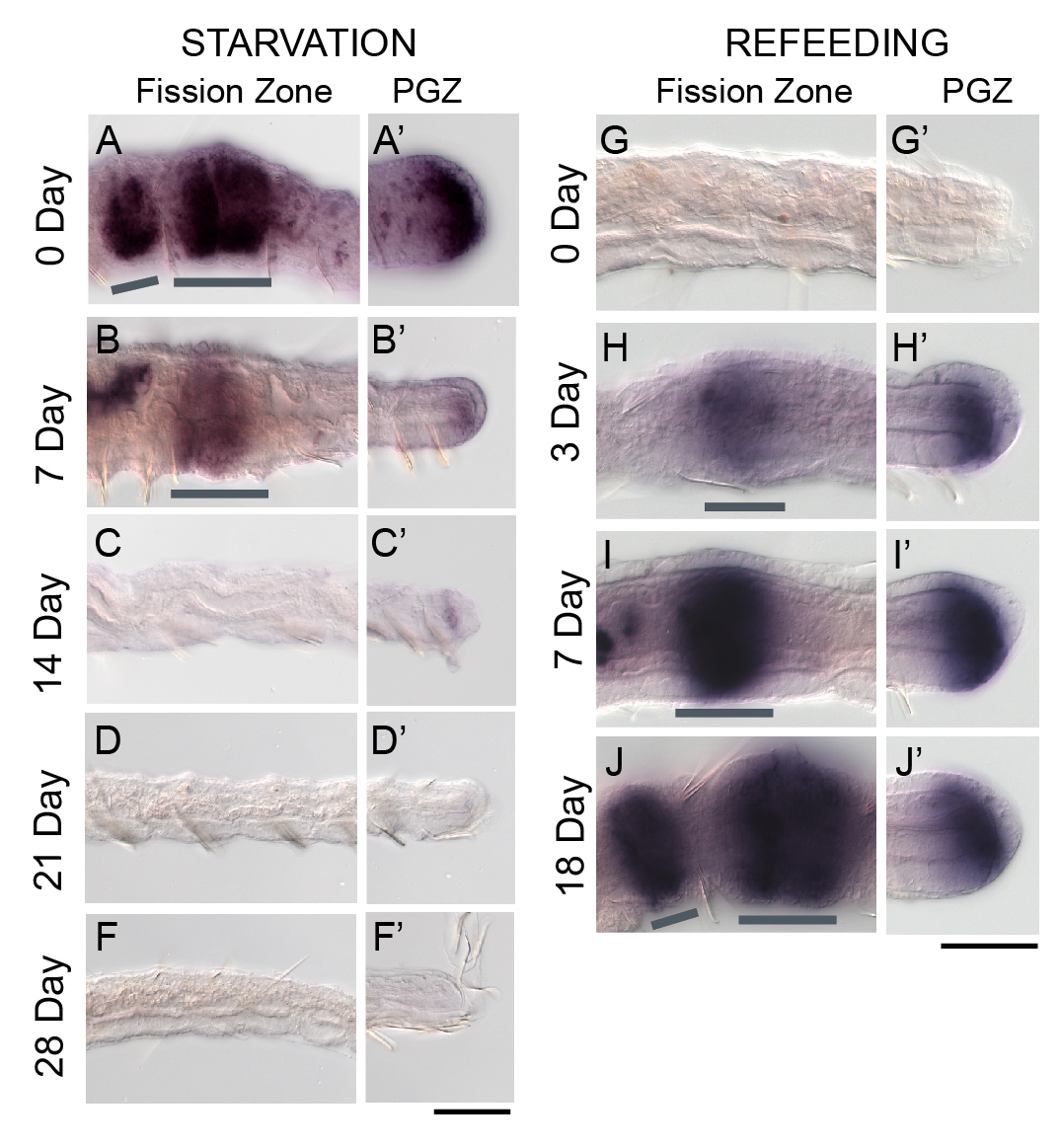


**Figure S2 – Effect of starvation and refeeding on *PRIle-vasa* expression.** (A-J') *PRIle-vasa* expression in the fission zone region (A-J) and posterior growth zone (A'-J') during starvation (A-F') and refeeding (G-J'). Expression decreases and becomes undetectable in both body regions as worms are starved over 28 days and is re-established in both body regions when starved worms are fed over 18 days. Fission zones are marked by gray bars. Note that animals in A and J possess multiple fission zones. Scale bars: 100 µm.


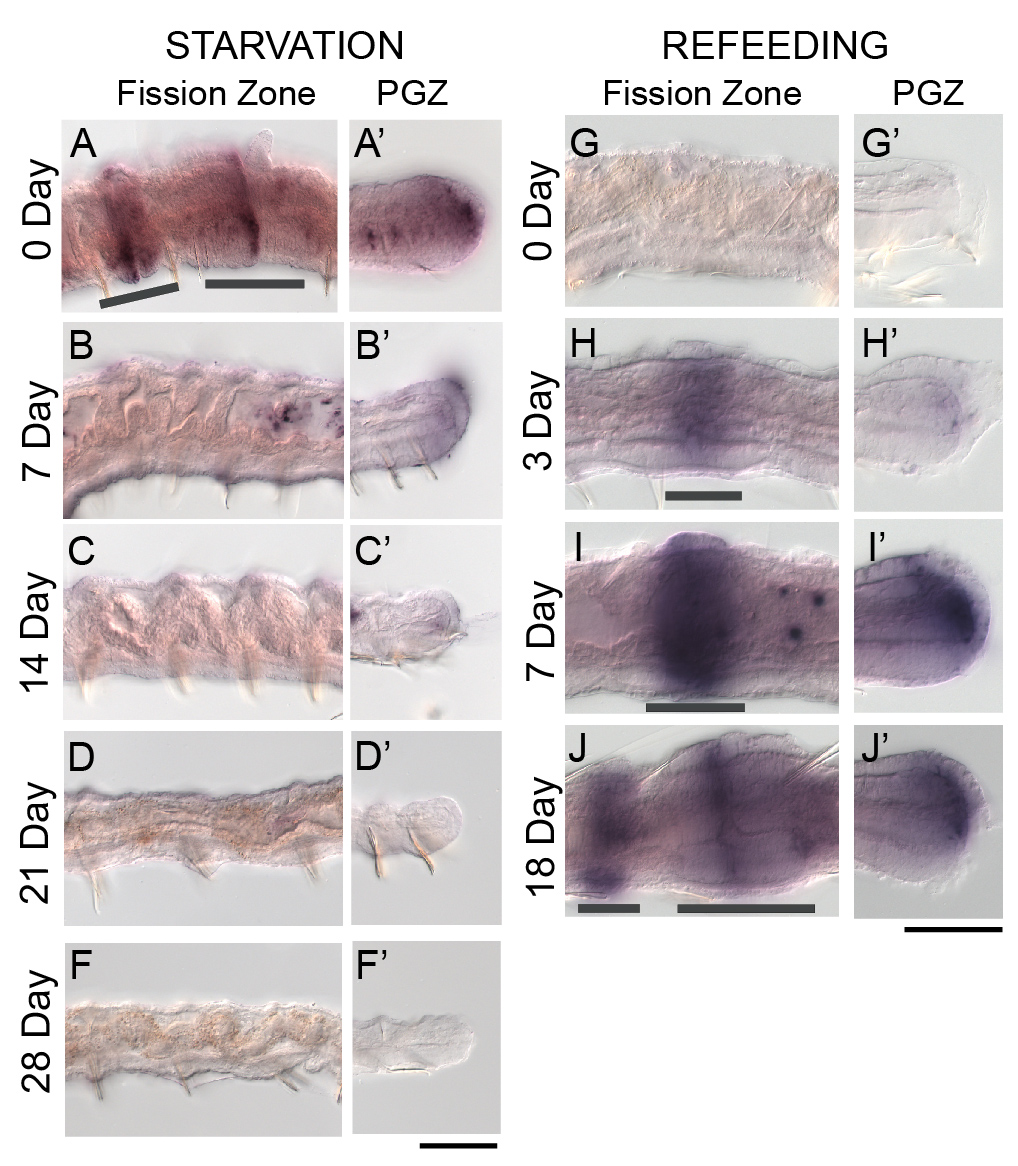

Supplement: Supplementary file 1 — 10.1186/s13227-016-0059-1 Effect of starvation and refeeding on PRIle-nanos and PRIle-vasa expression. [file 13227_2016_59_MOESM1_ESM.docx]
